# CAR-iNKT cells targeting clonal TCRVβ chains as a precise strategy to treat T cell lymphoma

**DOI:** 10.3389/fimmu.2023.1118681

**Published:** 2023-03-02

**Authors:** Aileen G. Rowan, Kanagaraju Ponnusamy, Hongwei Ren, Graham P. Taylor, Lucy B. M. Cook, Anastasios Karadimitris

**Affiliations:** ^1^Section of Virology, Department of Infectious Disease, Imperial College London, London, United Kingdom; ^2^Hugh and Josseline Langmuir Centre for Myeloma Research, Centre for Haematology, Department of Immunology and Inflammation, Imperial College London, London, United Kingdom; ^3^National Centre for Human Retrovirology, Imperial College Healthcare NHS Trust, St Mary’s Hospital, London, United Kingdom; ^4^Department of Haematology, Hammersmith Hospital, Imperial College Healthcare National Health Service (NHS) Foundation Trust, London, United Kingdom

**Keywords:** T cell lymphoma, adult T cell leukaemia/lymphoma, ATL, human T cell leukaemia virus type-1, human T cell lymphotropic virus type-1 (HTLV-1), chimeric antigen receptor (CAR) T-cells, iNKT, T cell receptor

## Abstract

**Introduction:**

Most T cell receptor (TCR)Vβ chain-expressing T cell lymphomas (TCL) including those caused by Human T cell leukaemia virus type-1 (HTLV-1) have poor prognosis. We hypothesised that chimeric antigen receptor (CAR)-mediated targeting of the clonal, lymphoma-associated TCRβ chains would comprise an effective cell therapy for TCL that would minimally impact the physiological TCR repertoire.

**Methods:**

As proof of concept, we generated CAR constructs to target four TCRVβ subunits. Efficacy of the CAR constructs was tested using conventional T cells as effectors (CAR-T). Since invariant NKT (iNKT) cell do not incite acute graft-versus-host disease and are suitable for ‘off-the-shelf’ immunotherapy, we generated anti-TCRVβ CAR-iNKT cells.

**Results:**

We show that anti-TCRVβ CAR-T cells selectively kill their cognate tumour targets while leaving >90% of the physiological TCR repertoire intact. CAR-iNKT cells inhibited the growth of TCL *in vivo*, and were also selectively active against malignant cells from Adult T cell leukaemia/lymphoma patients without activating expression of HTLV-1.

**Discussion:**

Thus we provide proof-of-concept for effective and selective anti-TCRVβ CAR-T and -iNKT cell-based therapy of TCL with the latter providing the option for ‘off-the-shelf’ immunotherapy.

## Introduction

Human T cell leukaemia virus type-1 (HTLV-1)-associated-adult T cell leukaemia/lymphoma (ATL), and the majority of other peripheral T cell lymphomas (TCL), have a dismal prognosis with current treatments. Carriers of HTLV-1, a virus that is endemic in many parts of the world, have a 5% risk of developing ATL in their lifetime (1, 2).

Expression of cell surface T-cell receptor (TCR)α/β chain heterodimers is restricted to normal and malignant T-cells. There are 22 functional TCRVβ subunit families in humans with each family comprising 0.5-9% (TCRVβ2 being the most frequent) of the normal T-cell repertoire. Signals from the TCR determine cell fate in normal T cells, and components of the TCR signalling pathway are frequently mutated in TCL including ATL, indicating a driving role for the TCR in TCL oncogenesis. Expression of surface TCR is stable in blood and lymph node lymphoma T cells (3–5). We and others have shown that TCL and ATL are clonal in nature with tumour cells sharing expression of a single TCRVβ subunit, with no bias in the usage of TCRVβ subunit families (3–5). Therefore, targeting the specific TCRVβ subunit expressed by TCL in a clonal manner would comprise a highly selective and tumour-specific therapeutic approach with ‘on target-off tumour’ toxicity limited to <9% of normal T-cells.

CAR-T therapeutic approaches in B cell leukaemias and lymphomas targeting pan-B cell markers such CD19 and CD20 can induce sustained clinical remissions in up to 40% of patients with relapsed/refractory disease, at the expense of clinically tolerable pan-B-cell depletion and hypoglobulinaemia (6). However, pan-T cell depletion would result in profound immunosuppression that is not clinically tolerable. Since most TCL are CD4+, anti-CD4 CAR-T treatment has also been proposed for these lymphomas (NCT03829540). Unless rescued by a stem cell transplant, such an approach would also result in immunosuppression akin to acquired immunodeficiency syndrome. Other CAR targets include CD5 and CD7; however, these are widely expressed on healthy T cells and can cause fratricide unless CAR effector cells are additionally edited to lack expression of these markers (7). Recently, efficacy using CAR-T cells to target the TCRβ chain constant region 1 which is expressed in ~40-60% of TCRαβ lymphocytes was reported (8). It remains to be seen what the impact of depleting 40-60% of the T cell repertoire will have on an individuals’ immune status. It may be that as in murine models, loss of half of the TCR sequences significantly impairs immunity in humans (9).

Autologous CAR-T immunotherapy can be limited by financial and logistical challenges and sub-optimal fitness of patient-derived T cells. To circumvent these challenges, we and others have developed CAR-iNKT cells as an ‘off-the-shelf’ allogenic immunotherapy. iNKT cells are a rare (<0.1% of T-cells), evolutionarily conserved subset of T cells which share features of innate and adaptive immune responses (10–12). In humans, iNKT are characterised by the expression of an invariant TCRVα24Jα18 chain which pairs with diverse TCRVβ11 chains (iTCR) (13). iNKT cells are restricted by CD1d, a non-polymorphic, glycolipid-presenting HLA class I-like molecule expressed on monocytes, macrophages dendritic cells, B cells, thymocytes and some epithelial tissues (14). In addition, iNKT cells have a memory effector phenotype and can migrate to extra-lymphoid tissues (15–17) where they modulate a variety of immune responses, including anti-tumour and anti-pathogen responses (18, 19). The anti-tumour effect of iNKT cells is further enhanced by their ability to eliminate tumour-associated macrophages on a CD1d-dependent manner (20).

Since iNKT cells protect against acute graft-versus-host disease (aGVHD) (21–24), CAR-iNKT-cell immunotherapy can be sourced from allogeneic healthy donors as ‘off-the shelf’ treatment without need for deletion of the endogenous TCR as is the case with conventional T cells.

Based on the above considerations, we explored targeting of ATL/TCL-specific TCRVβ chains through CAR engineering of T and iNKT cells.

## Materials and methods

### Patient samples

The clinical samples studied were donated, after written informed consent, by patients infected with HTLV-1 and attending the National Centre for Human Retrovirology (Imperial College Healthcare NHS Trust, St Mary’s Hospital, London). Research involving these samples from patients with ATL was conducted under the governance of the Communicable Diseases Research Group Tissue Bank, approved by the UK National Research Ethics Service (09/H0606/106, 15/SC/0089, 20/SC/0226). Details of the patient samples can be found in **Supplementary Table 1**.

### Cell lines and chemicals

HEK 293T were passaged twice a week by trypsinisation and maintained at 30-70% confluency in complete DMEM [Dulbecco’s modified eagle medium (DMEM) with 10% (v/v) Foetal bovine serum (FBS), 100 units/ml penicillin, 100 µg/mL streptomycin and 2mM L-glutamine]. JRT3-T3.5 were obtained from the ATCC and maintained at 1x10^5^- 1x10^6^ cells/ml in complete RPMI (RPMI with 10% (v/v) FBS, 100 units/ml penicillin, 100 µg/mL streptomycin and 2mM L-glutamine or iNKT) medium (RPMI with 10% FBS, 15 mM Hepes, 1 mM Sodium Pyruvate, 1x MEM nonessential amino acids, 4 mM L-Glutamine, 0.05 mM beta-mercaptoethanol, 100 units/ml penicillin and 100 µg/mL streptomycin). C1R-CD1d cells (25) were maintained at 1x10^5^- 1x10^6^ cells/ml in complete RPMI.

### Generation of CAR constructs

Four monoclonal antibodies were identified for proof of principle studies targeting each of TCR Vβ1 (clone BL37.2), Vβ2 (MPB2D5), Vβ9 (FIN9) and Vβ11 (C21) purchased unconjugated from Beckman Coulter. The amino acid sequence of the variable region of the heavy and light chains of BL37.2, MPB2D5 and FIN9 were determined by mass spectrometry (Rapid Novor, Canada). For clone C21, total RNA was extracted from hybridoma cells, reverse transcribed, and heavy and light chain fragments were amplified using isotype-specific primers. Amplified fragments were cloned into a standard cloning vector, transformed into E. coli, and five colonies from each region of interest were sequenced by Sanger sequencing (GenScript). Codon optimised gene fragments were synthesised by Genewiz and cloned into a lentiviral expression vector to generate the constructs shown in **Supplementary Figures 1, 2**.

### Production of lentiviruses

HEK293T cells were seeded at a density of 4 x 10^6^/10 cm plate in 10 ml complete DMEM. After 24h, cells were transfected with lentiviral expression vectors (**Supplementary Figure 2**) and second-generation packaging plasmids (psPAX2 and pMD2.G) using GeneJuice (Merck). Supernatants were collected 48h post-transfection and clarified by centrifugation at 500g for 5 minutes, and then passed through a 0.45 µM filter. Lentiviral particles were concentrated by ultracentrifugation at 23,000 rpm for 2h at 4°C, resuspended in 2-300 µl serum-free RPMI and stored at -80° C until use. The viral titre was determined by titration using HEK-293T cells.

### Isolation of peripheral blood mononuclear cells

Peripheral blood mononuclear cells (PBMCs) were purified from apheresis cones or EDTA anticoagulated blood by density gradient centrifugation. Briefly, cells were harvested from the cones and diluted in PBS up to 100 ml. 25 ml diluted cells were layered over 15 ml histopaque (Sigma) and centrifuged at 800g for 20 minutes. The buffy coat was harvested, washed with PBS (400g, 5 min at RT) and cryopreserved in FBS with 10% dimethyl sulfoxide (DMSO) until use.

### Lentiviral transduction of T and iNKT cells

PBMCs were thawed rapidly, washed twice in RPMI 10% FBS and resuspended in a small volume of media. iNKT were positively selected using magnetic bead separation according to the manufacturer’s instructions (Miltenyi Biotech), passing the cells over two consecutive LS columns. The purity of the positive fraction was evaluated by flow cytometric staining using a fixable viability stain (Live/Dead Near-Infrared, Life Sciences) and anti-TCRVα24-Jα18 BV421(clone 6B11), -TCRVB11 APC,-CD3 BV510, -CD4 BV605, -CD8 FITC. For iNKT cultures, the positively selected fraction was placed in culture with a 1:1 ratio of irradiated (35 Gy) autologous feeder cells prepared from the negative fraction. For T cell cultures, the iNKT-depleted flow through fraction was placed in culture alone. T and iNKT cells were cultured in iNKT media and stimulated with 50 ng/ml anti-CD3 and 50 ng/ml anti-CD28 (Miltenyi-Biotech) and 150 IU/ml IL-15. After 48h culture, the required volume of each lentivirus was placed in retronectin-coated plates to give a multiplicity of infection (MOI) of 2.5-5 infectious units per cell. Cells were harvested, counted and added to the transduction plate, then centrifuged at 1000g for 40 min at 32°C. On day 7 of iNKT cultures, CD1d-expressing feeder cells were loaded with 200 ng/ml α-Galactosylceramide (αGalCer, BioVision) for 2-4 h, irradiated and added to the culture at a ratio of 1:1. Cells were fed twice weekly with fresh media containing 150 IU/ml IL-15. If transduction efficiencies were below 50%, CAR expressing cells were enriched by staining with biotinylated protein-L followed by positive selection with streptavidin microbeads (Miltenyi Biotech).

### Quantification of lentiviral transduction efficiency

Transduction efficiency was evaluated from 5 days post-transduction. Cells were washed twice in cold PBS, incubated with 1 µg/ml biotinylated protein L (Pierce) for 45 min at 4°C. Cells were washed twice with PBS, stained with Live/dead near-infrared for 5 min at RT, washed with PBS 0.5% FBS and stained for 20 min at RT with streptavidin-PE/BV421 and anti-CD3, -CD4, -CD8, -TCRVβ11 and -TCRVα24-Jα18. Cells were acquired the same day using a BD Fortessa and analysed using Kaluza (Beckman Coulter).

### Isolation and culture of primary T cell targets

Autologous primary T cell lines expressing TCRVβ subunits of interest were established by magnetic enrichment and expansion *in vitro*. PBMC were stained for 20 min with PE-conjugated anti-TCRVβ antibodies (Beckman Coulter) at RT, washed once in complete RPMI and incubated for a further 20 minutes at 4° C with anti-PE microbeads (Miltenyi Biotech). After one further wash, cells were passed over two consecutive LS columns. The positive fraction was placed in culture with anti-CD3, anti-CD28 and 150 IU/ml IL-2.

### Generation of GFP-TCR – expressing JRT3-T3.5 cells

TCR β-chain sequences which contained Vβ subunits of interest were identified in publicly available databases. The codon optimised nucleotide sequence corresponding to the full-length beta chain was synthesised (Genewiz) and cloned into a third-generation lentiviral expression vector (LeGO-iG2).

### 7aad cytotoxicity assay

One day before the assessment of cytotoxicity, effector cells were counted and fed with fresh media. Target cells were washed twice with PBS, incubated in 0.5μM CFSE/CellTrace violet for 10 min at 37°C, and washed twice in iNKT media and placed back in culture with or without αGalCer as required. On the day of the assay, cells were centrifuged, resuspended in iNKT media with 15 IU/ml IL-15. Cells were mixed to achieve a range of effector:target (E:T) ratios, placed in duplicate in U bottomed 96 well plates, centrifuged for 1 min at 100g. Cells were cultured for 4-24h, centrifuged for 3 min at 800g and resuspended in PBS with 7aad (5 µg/ml). After 20 min incubation, each well was washed with 150 µl PBS, centrifuged for 3 min at 800g and resuspended in 100 µl PBS. In each well, the frequency of dead (7AAD+) target cells was enumerated by flow cytometry.

### Fresh PBMC cytotoxicity assay

One day before the assessment of cytotoxicity, effector cells were counted and fed with fresh media. On the day of the assay, cryopreserved PBMC were thawed were washed (400g for 5 min) once with iNKT media, once with PBS, incubated in 0.5µM CFSE/CellTrace violet for 10 min at 37°C, and washed twice in iNKT media. PBMC were placed in a U bottomed 96 well plate with 15 IU/ml IL-15 with and without CAR-iNKT cells at a range of effector:target ratios in triplicate. The plate was centrifuged for 1 min at 100g and cultured for 24h. Wells were washed with 150µl PBS (800g 3 min), stained with Live/dead near-infrared for 5 min at RT, washed with FACS buffer (PBS 7% (v/v) NGS) and stained with anti- TCRVβ-PE, -TCRαβ- FITC, -CD4-BV605, -CCR4-APC and -CD26-PeCy7 for 20 min at RT in FACS buffer. Cells were washed with 150 µl FACS buffer, fixed for 20 min with 150 µl fixation buffer (Biolegend) and washed once more with FACS buffer before storing at 4°C until acquisition.

### Intracellular cytokine staining and degranulation assay

Twenty four hours before evaluating intracellular cytokine production/degranulation, effector cells were fed, and target cells were stained with CFSE. On the day of the experiment, target and effector cells were harvested, counted and mixed to give a 1:1 E:T ratio in iNKT media containing 20 µg/ml DNase, 15 IU/ml IL-15, 10.6 µM brefeldin, 2 µM monensin (1x protein transport inhibitor, eBioscience) and 2.5 µl anti-CD107a-BV421 (clone H4A3)/200 µl culture well. Cells were cultured for 6 h, centrifuged at 800g for 3 minutes to remove the culture supernatant and resuspended in live/dead near infrared viability stain. After 5 minutes incubation at RT, 150 µl PBS 0.5% FBS was added to each well, and the plate was washed again. Cells were resuspended in 150 µl Fixation/Permeabilisation buffer (eBiosciences FoxP3 buffer set, Life Technologies) incubated for 30 min at RT and washed once in Permeabilisation buffer. Cells were stained with anti-CD3-BV510 (clone UCHT-1) -CD4-BV605 (RPA-T4) -CD8-AF700 (RPA-T8) -IFN-γ-BV711 (4S.B3), -TNF-α-PeCy7 (Mab11), Granzyme B-PE (QA16A02) and Perforin-APC (B-D48) diluted in Permeabilisation buffer. After 30 min incubation at RT, cells were washed once in 150 µl Permeabilisation buffer and resuspended in PBS 0.5% FBS until acquisition.

### Pentamer staining

HTLV-1-infected CD4+ cells express viral antigens on short term culture *ex vivo*, and thus could present the Tax11-19 peptide in the context of HLA-A*0201. Therefore, to minimise the possibility of downregulation of the cognate TCR on CTL due to antigen encounter during the culture period, CD4+ T cells were depleted from PBMCs from HLA-A*0201+ HTLV-1 carriers using anti-CD4 PE and anti-PE microbeads as described above. CD4-depleted PBMCs were stained with CellTrace violet. The positive and negative fractions were cultured in iNKT media with 15 IU/ml IL-15 in the presence or absence of CAR-iNKT cells. After 16-18h co-culture, cells were stained with live/dead near-infrared and resuspended in 40 µl PBS with 10 µl HTLV-1 Tax11-19 or Influenza A M158-66 Pentamer-APC for 10 minutes, after which anti-CD3-BV510 and -CD8-AF700 were added. After a further 20 min incubation at RT, cells were washed and fixed for 30 minutes by resuspending cells in 150 µl with Fixation buffer (Biolegend). After one wash with PBS, the frequency of live pentamer positive CellTrace violet+ CD8+ T cells in each culture was assessed by flow cytometry. Similarly, the CD4+ fraction was stained with live/dead, anti-CD4-BV605, -CD3-BV510, washed with PBS 7%NGS and fixed for 30 minutes with 150 µl eBioscience FoxP3 Fixation/Permeabilization buffer. Cells were then washed once in eBioscience Permeabilization buffer, and stained intracellularly with anti-Tax AF647 for 30 minutes. Cells were washed once more with 150 µl Permeabilization buffer, resuspended in PBS and stored at 4°C in the dark until acquisition. The number of Tax+CD4+CellTrace violet+ cells was measured by flow cytometry.

### *In vivo* experiment

*In vivo* work was performed under UK Home Office personal project license: PP8553679. 5x10^6^ TCRVβ2+ JRT cells were suspended in Matrigel and injected subcutaneously to the flank of NSG (NOD.Cg-*Prkdc^scid^ Il2rg^tm1Wjl^*/SzJ) mice obtained from Charles River Laboratories. On day 18, mice were randomised into three groups and received 1 x 10^6^ CAR-expressing iNKT cells/mouse by intravenous injection into the tail vein. Groups consisted of untreated (n=5), Vβ2 CAR-iNKT (n=7) or CD19 CAR-iNKT cells (n=5). Tumour volume for each group was measured periodically using calipers and tumour volumes were calculated using the standard formula (V=½ (Length × Width^2^)). At the end of the experiment, tumours were excised and weighed.

## Results

### Generation of CAR- constructs targeting TCRVβ1, -2, -9 and -11

We generated lentiviral CAR constructs to target TCRVβ1 (expressed on 3.5% of CD3+ T cells in healthy donors), -Vβ2(8.3%), -Vβ9 (3.2%) and -Vβ11 (1%). Since hybridoma clones were not available for the first three mAb, we employed reverse engineering of CARs after determining the amino acid sequence of mAbs by mass spectrometry. The corresponding nucleotide sequence was codon optimised for expression in human cells and used to construct second-generation CARs in which the ectodomain comprises CD8α leader peptide-VL-(GGGGS)_2/3_ linker-VH-CD8α hinge-CD8α transmembranous domain, while the endodomain is composed of the signalling domains of CD28-CD3ζ (**Supplementary Figure 1A**). To generate an anti-TCRVβ11 CAR with codon optimised VH/VL sequence, we amplified the expressed VH and VL chains using mRNA extracted from the hybridoma C21 (13).

**Figure 1:**
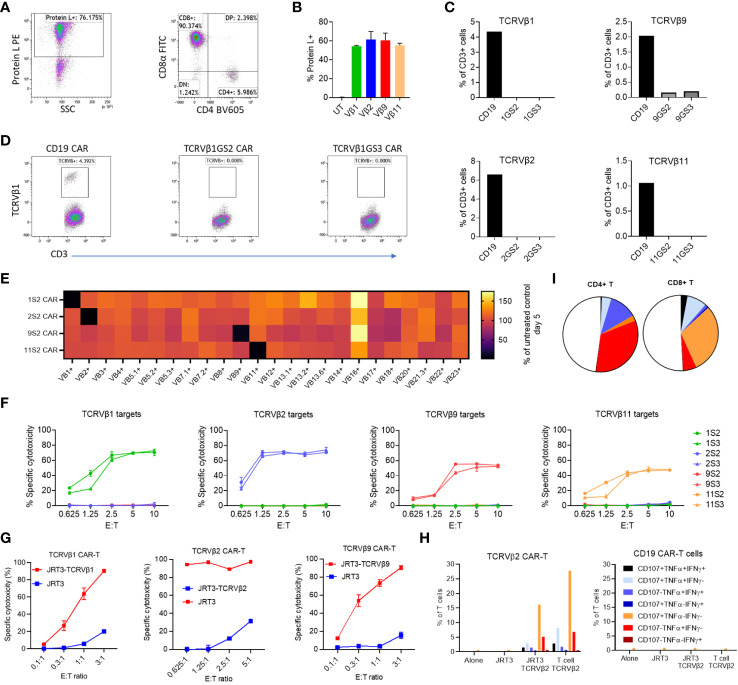
Efficacy and lack of significant off-target killing of primary T cells by Vβ-specific CAR-T cells. PBMC were transduced with anti-Vβ1, -2, -9, -11 or -CD19 CAR constructs. After three days the transduction efficiency was evaluated by protein L staining and the frequency of CD3+ T cells expressing each targeted Vβ subunit was quantified by flow cytometry. **(A)** Representative dot plots of CAR expression and phenotype of transduced cells. **(B)** Frequency of CAR-expressing cells in each culture. **(C)** Frequency of T cells expressing TCRVβ1, -2, -9 or -11 in transduced T cells. GS2 refers to the CAR construct containing two (G_4_S_1_) repeats and GS3 to the construct containing three (G_4_S_1_) repeats. **(D)** Representative dot plots showing TCRVβ1 staining in the same experiment. **(E)** PBMC from two normal donors were cultured alone or transduced with anti-Vβ1, 2, 9 or 11 CAR (GS2) constructs. Five days post transduction, transduction efficiency was evaluated by protein L staining, and the frequency of CD3+ T cells expressing each of 24 TCRVβ subunits was quantified. Values shown are the mean percentage of cells expressing each subunit normalised to the frequency of cells expressing that subunit the untransduced PBMC control from each individual. **(F)** Killing of *in vitro*-expanded primary T cell lines expressing TCRVβ1, 2, 9 or 11 by T cells transduced with anti-Vβ GS2/GS3 CAR constructs. Target cells were stained with CFSE co-cultured with effectors at a range of effector to target (E:T) ratios in duplicate. After 24h cells were harvested and stained with 7aad. The frequency of dead targets in each culture condition was assayed by flow cytometry. **(G)** Killing of a T lymphoblastic lymphoma cell line (JRT3) transduced to express TCR containing TCRVβ subunits. **(H)** Cytokine production and degranulation (CD107a mobilisation) by anti-Vβ2 CAR-T cells cultured alone, in the presence of TCRVβ2-transduced or untransduced JRT3-T3.5 cells or in the presence of primary T cells expressing TCRVβ2. **(I)** Representative data showing cytokine production and degranulation by CD4+ CAR-T and CD8+ CAR-T.

To begin testing the activity and specificity of CARs of interest we lentivirally transduced them into PBMC T cells of two healthy donors. Five days post-transduction, CAR expression on T cells was >50% (**Figures 1A, B**). At the same time, with reference to untransduced T cells, we quantified the frequency of T cells expressing each of 24 TCRVβ chains in CAR-transduced PBMC. We found that T cells expressing TCRVβ1, Vβ2, Vβ9 and Vβ11 were almost entirely depleted from PBMCs transduced with the respective cognate CAR construct (median 98% reduction; range 90-100%; **Figures 1C–E**; **Supplementary Figure 1B**). By contrast, we observed a 4% increase in the median frequency of untargeted Vβ subunits in transduced cultures relative to the untransduced control indicating lack of ‘off-target’ killing by TCRVβ family-specific CAR-T cells.

Thus, anti-TCRVβ1,-2, -9 and -11 CAR are robustly expressed, active and selective against their cognate TCRVβ chain targets.

### Anti-TCRVβ chain CAR against primary and cancer T cell lines

To further investigate the anti-T cell activity and TCRVβ specificity of the four CARs, we first tested TCRVβ CAR-T cells against expanded autologous primary T cell lines highly purified to express TCRVβ chains of interest (**Supplementary Figure 3A**). We found that all four anti-TCRVβ CAR-T cells selectively killed their cognate T cell line (**Figure 1F**). However, the level of cytotoxicity differed and reflected the intensity of staining with the corresponding mAb, with both being highest for anti-TCRVβ1 and β2 and lowest for TCRVβ9 and TCRVβ11 (**Figure 1F** and **Supplementary Figure 3A**).

Next, we engineered the JRT3-T3.5 T cell line to express the TCRVβ1 or 2 chains as targets. JRT3-T3.5 is a derivative of the Jurkat T cell line, itself derived from a patient with TCRVβ+ lymphoblastic T cell lymphoma. Due to deletion of the endogenous TCRVβ chain gene, JRT3-T3.5 cells lack surface expression of TCR-CD3 complexes. Introduction of an exogenous TCRVβ cDNA restored expression of TCR-CD3 complexes on the cell surface (**Supplementary Figure 3B**) at levels equivalent to primary T cells (**Supplementary Figure 3C**). Accordingly, we found that anti-TCRVβ1 & 2 CAR-T cells kill JRT3-T3.5 cells expressing their cognate target TCRVβ but not the parental cell line (**Figure 1G**).

In a complementary functional approach, we found that with intracellular staining, anti-TCRVβ2 CAR-T cells expressed IFNγ, TNFα and CD107a (denoting cytotoxic degranulation) when co-cultured with TCRVβ2-expressing JRT3-T3.5 cells or primary T cell line but not when cultured in the presence of the parental JRT3-T3.5 or alone, with >30% of CD4+ and CD8+ CAR-T cells being poly-functional, i.e. co-expressing at least two molecules (**Figures 1H, I**).

Together, these findings show that anti-TCRVβ CARs developed by reverse engineering are highly specific and active *in vitro*.

### Anti-TCRVβ CAR-iNKT cells for ‘off-the -shelf’ immunotherapy of T cell malignancies

We and others have demonstrated the feasibility of deploying iNKT cells as effectors for CAR immunotherapy of blood cancers.

**Figure 2:**
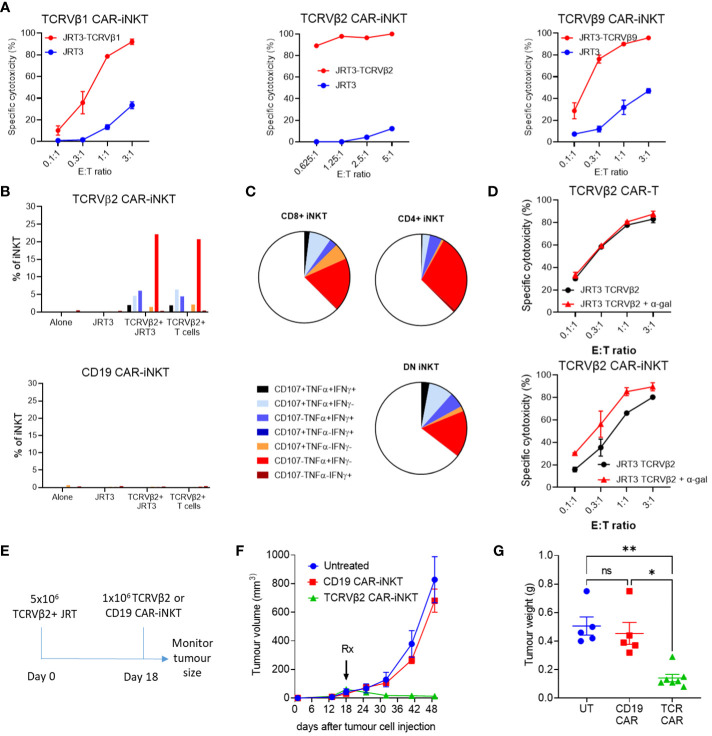
Cytotoxic activity of anti-TCRVβ CAR-iNKT *in vitro* and *in vivo*. **(A)** Killing of untransduced (JRT3) and TCR-GFP-transduced (JRT3-TCRVβ) JRT-3 T 3.5 cells by anti-Vβ CAR-iNKT cells. Target cells were stained with CFSE co-cultured with effectors at a range of effector to target (E:T) ratios in duplicate. After 24h cells were harvested and stained with 7aad. The frequency of dead targets in each culture condition was assayed by flow cytometry. Results are representative of two or more independent experiments. **(B)** CD107a mobilisation, IFN-γ and TNF-α production by CD3+ anti-Vβ2 and anti-CD19 CAR-iNKT when cultured alone or in the presence of untransduced JRT3-T3.5 cells (JRT), JRT3-T3.5 cells transduced with Vβ2+ TCR-GFP (Vβ+), or primary cells expressing Vβ2 (TCRVβ2+T cells). Results are from a single experiment and are representative of 2 independent experiments. **(C)** Representative data showing cytokine production and degranulation by CD4+, CD8+ and double negative CAR-iNKT. **(D)** Enhancement of killing of α-gal loaded TCRVβ2-GFP-transduced JRT3-T3.5 cells by anti-Vβ2 CAR-iNKT but not anti Vβ2 CAR-T cells. Where indicated, target cells were incubated with 200ng/ml α-gal before co-culture with effectors. Results shown are representative of two repeat experiments. **(E)** *In vivo* protocol. 5x10^6^ TCRVβ+ JRT cells were suspended in Matrigel and injected subcutaneously. On day 18, 1 x 10^6^ effector cells were injected intravenously into the tail vein. Groups consisted of untreated (n=5), Vβ2 CAR-iNKT (n=7) or CD19 CAR-iNKT (n=5). **(F)** Tumour volume for each group was measured periodically using calipers and **(G)** tumour weight was determined after the experiment was terminated. ** indicates p<0.001, * indicates P<0.05 and ns indicates no significant difference, Kruskal-Wallis test with Dunn's multiple comparisons test.

We therefore, using our established manufacturing protocol (25), generated anti-TCRVβ1, 2, 9 & 11 CAR-iNKT cells (**Supplementary Figures 4A–C**). As predicted, due to fratricidal effect directed against the TCRVβ11-expressing iNKT cells, viable anti-TCRVβ11 CAR-iNKT were not generated. Transduction efficiencies of iNKT cells were more variable than T cells, however CAR-iNKT could be readily purified to obtain >90% transduced cells (**Supplementary Figure 4D**). Anti-TCRVβ1,2&9 CAR-iNKT cells efficiently killed JRT3-T3.5 as well as primary T cell lines expressing the corresponding TCRβ chain (**Figure 2A**) while CD4+ and CD4- CAR-iNKT cells specifically expressed or co-expressed IFNγ, TNFα and CD107a upon stimulation with TCRVβ2-expressing JRT3-T3.5 and primary T cells (**Figures 2B, C**).

Since JRT3-T3.5 cells express CD1d (**Supplementary Figure 4E**), we tested whether reactivity of anti-TCRVβ CAR-iNKT cell could be enhanced in the presence of αGalCer, a glycolipid ligand presented by CD1d that powerfully and selectively activates iNKT-cells (26, 27). We found that while anti-TCRVβ1&2 CAR-T cytotoxicity against parental and cognate TCRVβ-expressing JRT3-T3.5 cells was not enhanced in the presence of αGalCer, cytotoxicity of anti-TCRVβ2 CAR-iNKT was enhanced against both targets and more so against the parental JRT3-T3.5 T cells (**Figure 2D**) thus indicating the functional relevance of iTCR in the anti-tumour activity of CAR-iNKT cells. In the absence of αGalCer, the efficiencies of CAR-T and CAR-iNKT were comparable (**Supplementary Figure 5**).

We further tested the activity of TCRVβ CAR-iNKT cells in an *in vivo* model of T cell lymphoma in which TCRVβ2-expressing JRT3-T3.5 T cells were injected subcutaneously to the flank of NSG mice (**Figure 2E**). After tumour engraftment, mice were left untreated or treated with i.v. transfer of 10^6^ TCRVβ2 or CD19 CAR-expressing iNKT cells per mouse. Consistent with lack of expression of CD19 by JRT3-T3.5 T cells, CD19 CAR-iNKT failed to impact the growth of T cell lymphoma tumours; by contrast, TCRVβ2 CAR-iNKT cells significantly inhibited tumour growth as measured by tumour volume and weight (**Figures 2F, G**).

Finally, consistent with previous work, CAR-iNKT cell-treated mice showed no evidence of weight loss or other clinical signs of aGVHD after 42 days of monitoring (**Supplementary Figure 4F**), thus supporting the notion of using allogeneic iNKT cells as an ‘off-the-shelf’ platform for immunotherapy of cancer without risk of aGVHD.

Together these data demonstrate the therapeutic potential of allogeneic TCRVβ CAR-iNKT cells for the treatment of T cell lymphomas.

### Anti-TCRVβ CAR-iNKT cells are active against ATL

**Figure 3:**
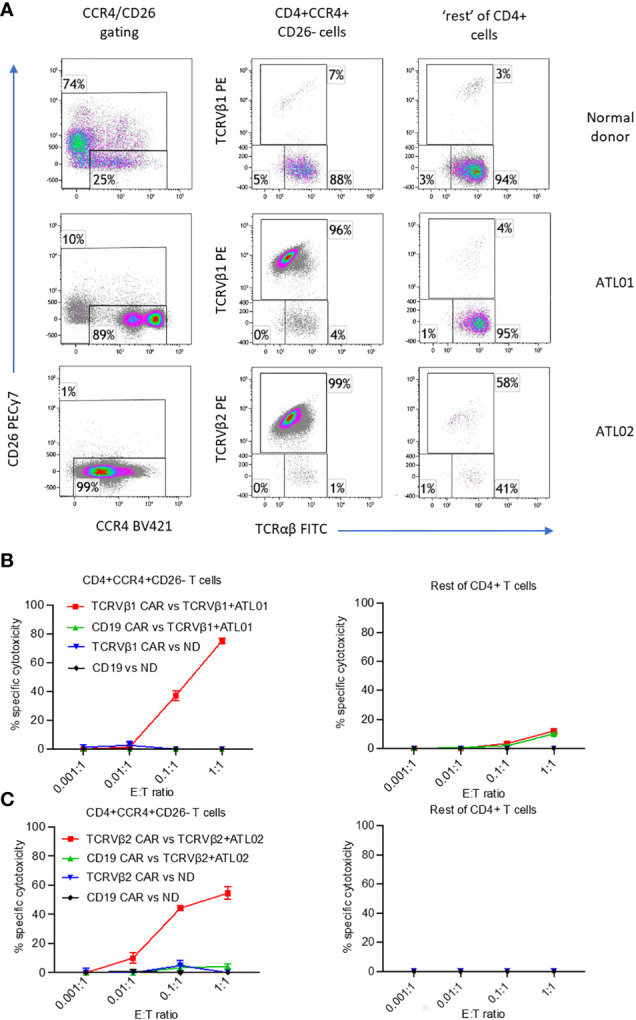
Killing of *ex vivo* CD4+CCR4+CD26- ATL cells expressing TCRVβ1 and Vβ2 by anti-TCRVβ CAR-iNKT. Cryopreserved PBMC from two ATL patients and normal donor (ND) were stained with CellTrace violet and co-incubated with anti-Vβ1 -Vβ2 or-CD19 CAR-iNKT at the indicated effector:target (PBMC) ratios in triplicate. **(A)** Immunophenotype of samples before co-culture. After 24h co-culture, cells were stained with a viability stain, anti-CD4, CCR4, CD26, TCRVβ1 or 2 and TCRαβ, fixed, and the frequency of dead CD4+CCR4+CD26- T cells and CCR4- and CCR4+CD26+ cells (‘rest’ of CD4+ T cells) was determined by flow cytometry. **(B)** Killing of cells from patient ATL01 and the normal donor by TCRVbeta1 CAR-iNKT. **(C)** Killing of cells from patient ATL02 and the normal donor by TCRVbeta2 CAR-iNKT.

As well as against JRT3-T3.5 T cell lymphoma cells, we also tested the reactivity of allogeneic anti-TCRVβ1&2 CAR-iNKT cells against PBMC from a normal donor and against PBMCs from two patients with ATL who had high frequencies of circulating leukemic cells which clonally express TCRVβ1 or TCRVβ2 (**Figure 3A**). In addition to their clonal TCR, as we previously demonstrated (5), ATL malignant cells also co-express CD4 and CCR4 and are negative for CD26 (**Figure 3A**; **Supplementary Figure 6**). Therefore, we co-cultured anti-CD19 CAR-iNKT and matched anti-TCRβ CAR-iNKT cells with PBMC from each ATL and normal donor at a range of E:T ratios. Because co-culture with the anti-TCRVβ CAR-iNKT cells could block subsequent staining with the same anti-TCRVβ antibody clone, we used the immunophenotype CD4+CCR4^med/hi^CD26- to identify the lymphoma/leukaemia cells in PBMC from patients with ATL. We found that in patients 1 and 2, CCR4+CD26- cells comprised 90% of CD4+ cells and nearly all expressed TCRVβ1 or TCRVβ2 respectively, while in the normal donor, CCR4+CD26- cells comprised 25% of CD4+ cells and TCRVβ1 and TCRVβ2 comprised <8%.

At an Effector : CD4+ T cell ratio of 1:1, anti-TCRVβ1 CAR-iNKT killed 75% of CD4+CCR4+CD26- cells in patient 1, and 10% of the ‘rest’ (i.e., not CCR4+CD26-) of CD4+ cells (**Figure 3B**). At the same ratio in patient 2, anti-TCRVβ2 CAR-iNKT killed 54% of CD4+CCR4+CD26- cells (**Figure 3C**). The frequency of CD4+ cells expressing other TCRVβ subunits was too low to determine whether there was no off-target killing of normal CD4+ cells in this patient. Minimal killing of CD4+CCR4+CD26- cells and ‘other CD4+’ cells was observed when PBMC from patients or the normal donor were co-cultured with anti-CD19 CAR-iNKT cells. Similarly, minimal killing was observed after PBMC from the normal donor were co-cultured with anti-TCRVβ1 and -TCRVβ2 CAR-iNKT cells, likely because of the low frequency of target cells present in the populations analysed (**Figures 3B, C**).

We conclude that anti-TCRVβ CAR-iNKT cells are highly active and specific against primary ATL cancer cells.

### Effect of anti-TCRVβ CAR-iNKT on antiviral CTL immunity and HTLV-1 proviral gene expression

**Figure 4:**
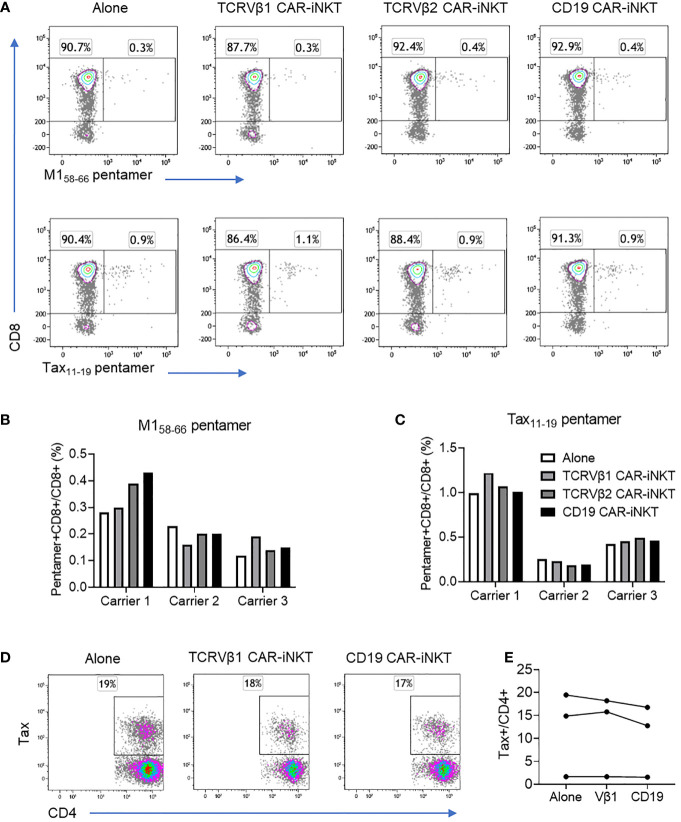
Effect of CAR-iNKT on the frequency of antiviral CTL and HTLV-1 expressing CD4+ T cells. **(A–C)** Frequency of M1_58-66_ and Tax_11-19_ HLA-A*0201 pentamer+CD8+ T cells after co-culture with CAR-iNKT. CD4-depleted PBMC from three HLA-A*0201+ HTLV-1 carriers were stained with CellTraceViolet and were cultured alone or with an 1:1 ratio of anti-TCRVβ1 -Vβ2 or -CD19 CAR-iNKT. After 16-18h co-culture, cells were stained with a viability stain, and anti-CD3, -CD8 antibodies and M1_58-66_ or Tax_11-19_ pentamers. Cells were analysed by flow cytometry and the frequency of live Pentamer+CellTraceViolet+CD3+CD8+ cells was determined. **(D, E)** Frequency of HTLV-1 Tax expressing CD4+ cells after co-culture with CAR-iNKT. CellTraceViolet stained, positively selected CD4+ cells from the same donors were cultured alone or in the presence of Vβ1- Vβ2- or CD19-CAR iNKT. After 16-18h, cells were stained with a viability stain, anti-CD3,-CD4, -CD8 and -TCRVβ1 or -TCRVβ2. Cells were subsequently fixed, and stained intracellularly with anti-Tax antibody. Cells were analysed by flow cytometry and the frequency of live TCRVβ1/2+CD3+ cells and live Tax+CD4+ cells were determined.

In order to further demonstrate selective targeting we studied the *in vitro* impact of anti-TCRVβ CAR-iNKT cells on antiviral CD8+ T cells in HTLV-1-infected individuals. For this purpose, we cultured PBMC from three HLA-A*0201+ HTLV-1-infected individuals alone or in the presence of CAR-iNKT and evaluated the frequency of T cells which bound HLA-A*0201 HTLV-1 Tax_11-19_ or Influenza A M1_58-66_ peptide-MHC pentamers (**Figures 4A, B** and **Supplementary Figure 7A**). When cultured alone, the frequency of Influenza A M1_58-66_ pentamer+ CD8+ T cells ranged from 0.12-0.28% of CD3+ cells, and the frequency of HTLV-1 Tax_11-19_ pentamer+ CD8+ T cells ranged from 0.25-0.99%. When co-cultured with Vβ1-, Vβ2- or CD19- CAR-iNKT cells, there was no significant change in the frequency of pentamer+ cells (**Figures 4A–C**). Efficient depletion of T cells expressing TCRVβ1 and -β2 by their cognate CAR-iNKT was confirmed in parallel cultures from the same donors (**Supplementary Figure 7**).

Since killing of target cells by CAR-iNKT is associated with secretion of inflammatory cytokines we asked whether killing by CAR-iNKT would enhance spontaneous expression of HTLV-1 Tax protein, a process that might facilitate infectious spread of HTLV-1 infection through triggering virus particle production. For this purpose, positively selected CD4+ T cells from the same three HTLV-1-infected individuals were first cultured alone or in the presence of anti-TCRVβ1, -Vβ2 and -CD19 CAR-iNKT cells for 18 hrs followed by evaluation by intracellular staining of the frequency of Tax-expressing CD4+ T cells. Despite clear evidence of CAR-mediated depletion of T cells expressing the target TCRVβ molecules, there was no change in the frequency of Tax+CD4+ T cells in any of the CAR-iNKT co-cultures when compared with cells cultured alone (**Figures 4D, E**).

Therefore, CAR-iNKT cells targeting TCRVβ chains do not impair CTL immunity against viral antigens, neither do their promote replication activity of HTLV-1, both important considerations for the safe treatment of TCL and ATL.

## Discussion

This study, in conjunction with recent work (28, 29), provides proof-of-concept that anti-TCRVβ CAR immunotherapy can be developed as a sensitive, specific and highly effective strategy for the treatment of TCRVβ-expressing T cell lymphomas.

Given that the distribution of the TCRVβ repertoire in clonally expanded T cell lymphoma cells is the same as in normal T cells, approximately 15% of TCL can be targeted by the three CARs developed and tested here, thus paving the way for development of CARs against all TCRVβ families for which mAbs are available (currently for ~70% of TCRβ chain families) or can be developed. Of the three TCRVβ CARs, best *in vitro* killing was observed against TCRVβ1 and 2- and less so for TCRVβ9-expressing targets. This pattern correlated with the intensity of staining of T cells by the corresponding mAbs, suggesting that the anti-TCRVβ9 mAb and thus the corresponding CAR are of lower affinity, highlighting the need to select high affinity mAb.

The robust anti-TCL activity of anti-TCRVβ CARs was shown using two effector platforms: T and iNKT cells thus offering the prospect of either autologous or allogeneic, ‘off-the-shelf’ cellular immunotherapy respectively. The subcutaneous TCL model used to test the *in vivo* efficacy of TCRVβ CAR-iNKT cells is faithful to human TCLs in which skin is often a primary or secondary site of disease. Although potential toxicity of allogeneic CAR-iNKT cells will be ultimately determined in clinical trials, early clinical experience with allogeneic CAR-iNKT cells against B cell lymphoma suggested absence of significant toxicity and aGVHD (30, 31). Our data also suggest that CAR-iNKT cells would offer additional therapeutic advantages over CAR-T cells in TCRVβ and CD1d co-expressing cases of T cell malignancies such as T lymphoblastic lymphoma, as exemplified by the Jurkat T cell line. Incorporation of αGalCer in the therapeutic armamentarium could result in further enhancement of the CAR-iNKT cell-mediated anti-leukaemia/lymphoma effect, although this should be tested in *in vivo* future studies. Further, the allogeneic CAR-iNKT cell strategy lack the potential for tumour CAR target masking. Indeed, use of autologous T cells risks CAR transduction of tumour cells which, as previously described for CD19 CAR-T cells in B cell lymphoma (32), might mask the target TCRVβ chain resulting in tumour escape. In the case of ATL, use of allogeneic iNKT cells as opposed to autologous T cells, has the additional advantage of providing HTLV-1-free effector cells.

Reassuringly, anti-TCRVβ CAR-iNKT immunotherapy does not appear to impact adaptive anti-viral immunity: there was no change in the frequency of Influenza A-specific or HTLV-1 Tax-specific CD8+ T cells when PBMC from HTLV-1 carriers were cultured with and without anti-TCRVβ CAR-iNKT. We also evaluated the effect of CAR-iNKT activity on proviral expression by by-standing HTLV-1 infected CD4+ T cells and observed no difference in HTLV-1 Tax expression in the presence or absence of CAR-iNKT cells. Thus, at least in short term *in vitro* assays, we have not observed any bystander activation provrial gene expression in HTLV-1 infected T cells that favours infectious spread of HTLV-1.

We envisage that the precision medicine approach for the treatment of TCL and ATL we describe here will expand to involve anti-TCRVβ CARs for most if not all TCRVβ chains. However, this poses the challenge of clinical development including safety testing for each of these CARs. We expect that this will be best achieved by using the allogeneic iNKT cell platform which will allow the swift deployment of pre-manufactured allogeneic anti-TCRVβ CAR-iNKT cells in HTLV-1 endemic areas and in combination with advanced clinical trial designs such as Bayesian optimal interval design (33). Such ‘off-the-shelf’, anti-TCRVβ CAR-iNKT cells can be tested not only in patients with ATL but also potentially in HTLV-1-infected individuals in whom, as we recently showed, clonal T cell expansions above a certain threshold portend high risk of progression to ATL, a frequently incurable cancer (34, 35).

In summary, our work provides the rationale for clinical development of anti-TCRVβ CAR-iNKT cells as an effective and highly selective “off-the-shelf” immunotherapy for the largely incurable T cell malignancies.

## Data availability statement

The raw data supporting the conclusions of this article will be made available by the authors, without undue reservation.

## Ethics statement

The clinical samples studied were donated by patients infected with HTLV-1 and attending the National Centre for Human Retrovirology (Imperial College Healthcare NHS Trust, St Mary’s Hospital, London), where written informed consent was obtained. Research involving these samples from patients with ATL was conducted under the governance of the Communicable Diseases Research Group Tissue Bank, approved by the UK National Research Ethics Service (09/H0606/106, 15/SC/0089, 20/SC/0226). The animal study was reviewed and approved by the UK Home Office personal project license: PP8553679.

## Author contributions

Study conception and design, AR and AK; experimental work, AR and KP; provision of reagents, HR; patient recruitment and characterisation, LC and GT; drafting manuscript AR and AK, editing manuscript, all authors. All authors contributed to the article and approved the submitted version.
