# Supplementary material for: CAR-iNKT cells targeting clonal TCRVβ chains as a precise strategy to treat T cell lymphoma
Source: Front Immunol. 2023 Mar 2;14:1118681. doi: 10.3389/fimmu.2023.1118681 (PMC10019783; doi:10.3389/fimmu.2023.1118681)
Supplement: Supplementary file 1 [file Presentation_1.pptx]

## Slide 1
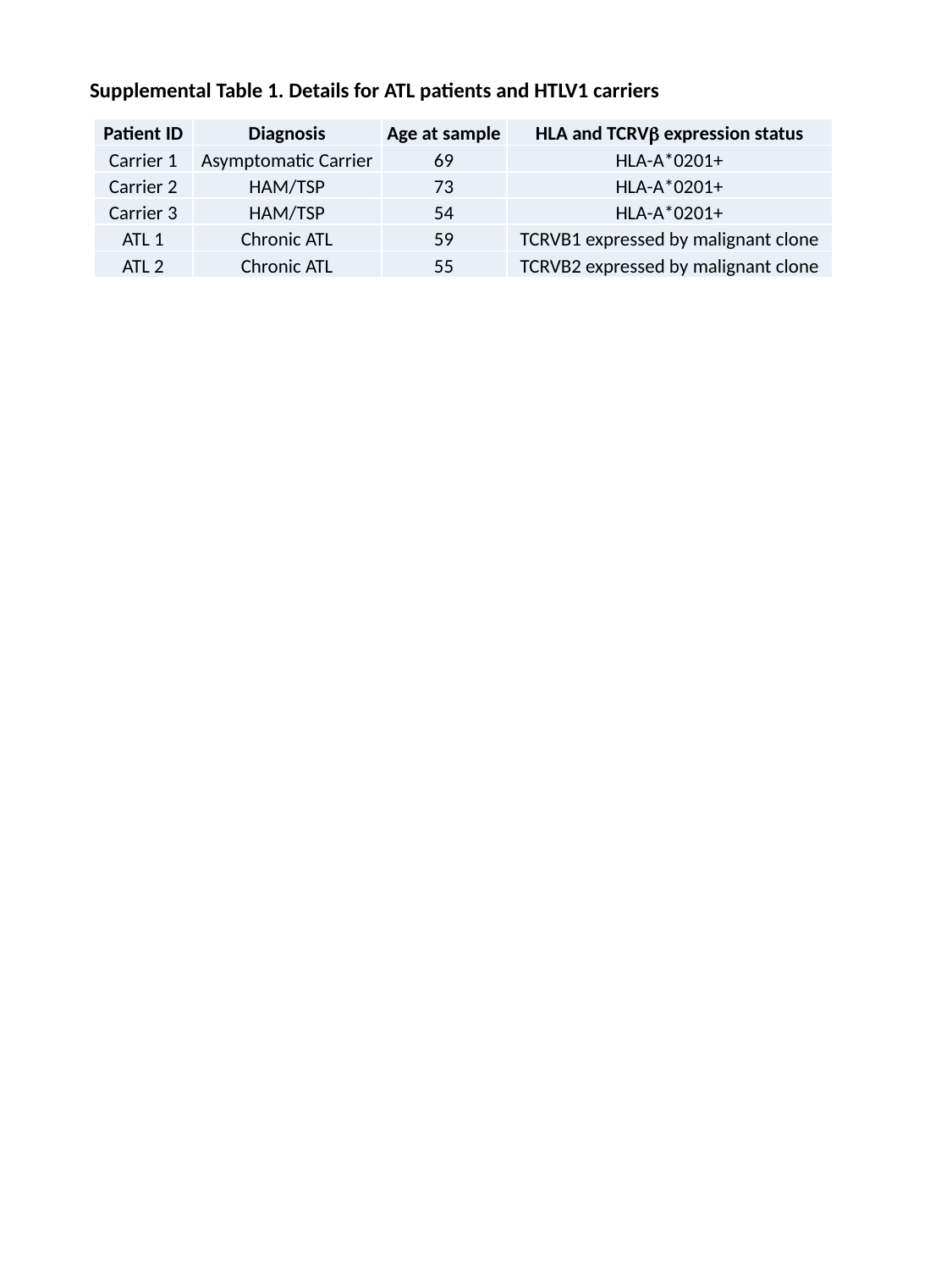

Supplemental Table 1. Details for ATL patients and HTLV1 carriers
| Patient ID | Diagnosis | Age at sample | HLA and TCRVb expression status |
| --- | --- | --- | --- |
| Carrier 1 | Asymptomatic Carrier | 69 | HLA-A\*0201+ |
| Carrier 2 | HAM/TSP | 73 | HLA-A\*0201+ |
| Carrier 3 | HAM/TSP | 54 | HLA-A\*0201+ |
| ATL 1 | Chronic ATL | 59 | TCRVB1 expressed by malignant clone |
| ATL 2 | Chronic ATL | 55 | TCRVB2 expressed by malignant clone |

## Slide 2
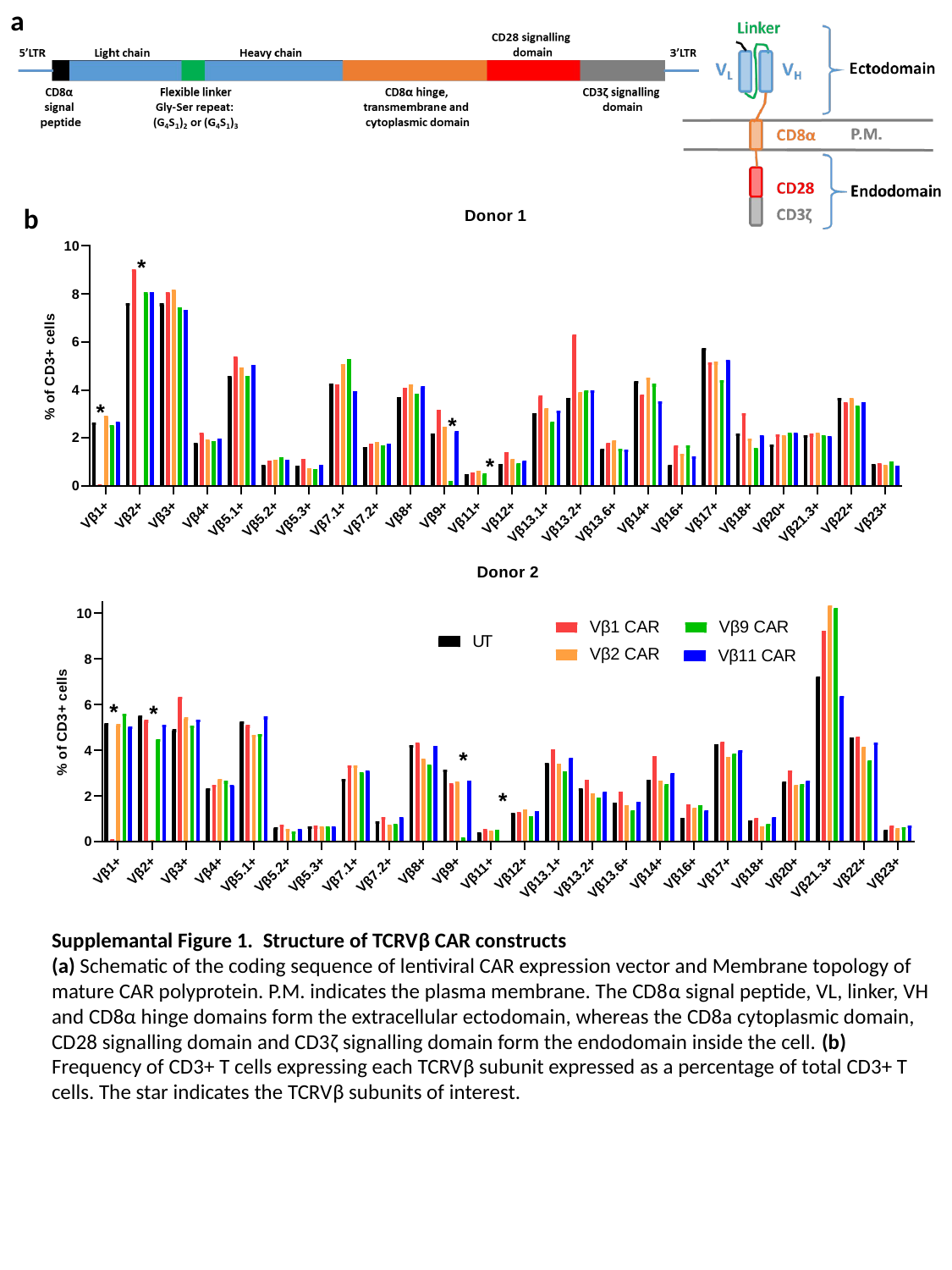

a
b
Supplemantal Figure 1. Structure of TCRVβ CAR constructs
(a) Schematic of the coding sequence of lentiviral CAR expression vector and Membrane topology of mature CAR polyprotein. P.M. indicates the plasma membrane. The CD8α signal peptide, VL, linker, VH and CD8α hinge domains form the extracellular ectodomain, whereas the CD8a cytoplasmic domain, CD28 signalling domain and CD3ζ signalling domain form the endodomain inside the cell. (b) Frequency of CD3+ T cells expressing each TCRVβ subunit expressed as a percentage of total CD3+ T cells. The star indicates the TCRVβ subunits of interest.

## Slide 3
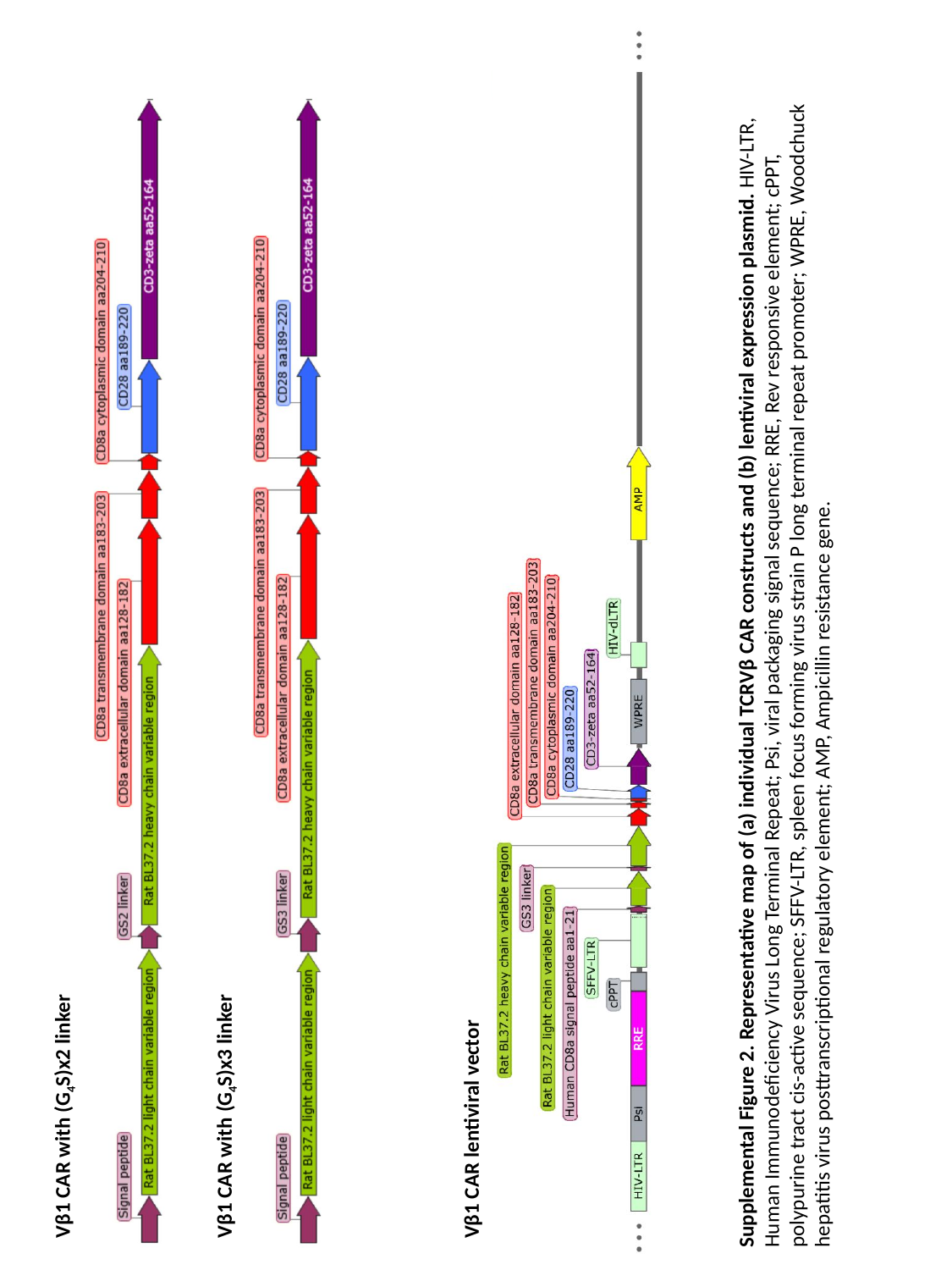

Supplemental Figure 2. Representative map of (a) individual TCRVβ CAR constructs and (b) lentiviral expression plasmid. HIV-LTR, Human Immunodeficiency Virus Long Terminal Repeat; Psi, viral packaging signal sequence; RRE, Rev responsive element; cPPT, polypurine tract cis-active sequence; SFFV-LTR, spleen focus forming virus strain P long terminal repeat promoter; WPRE, Woodchuck hepatitis virus posttranscriptional regulatory element; AMP, Ampicillin resistance gene.
Vβ1 CAR with (G4S)x2 linker
Vβ1 CAR with (G4S)x3 linker
Vβ1 CAR lentiviral vector

## Slide 4
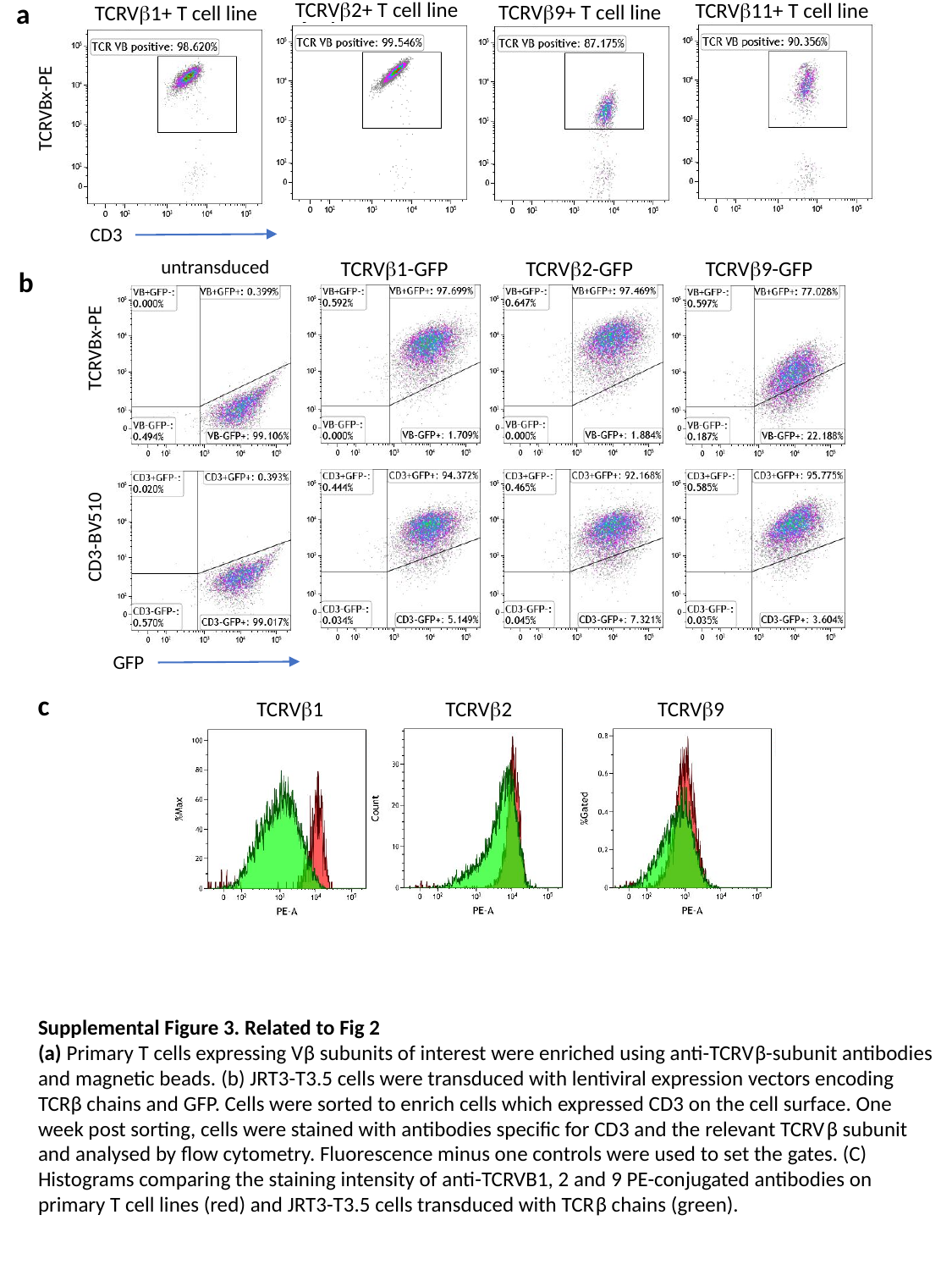

TCRVb2+ T cell line
TCRVb11+ T cell line
TCRVb9+ T cell line
TCRVb1+ T cell line
TCRVBx-PE
CD3
a
untransduced
TCRVb1-GFP
TCRVb2-GFP
TCRVb9-GFP
TCRVBx-PE
CD3-BV510
GFP
b
c
TCRVb2
TCRVb9
TCRVb1
Supplemental Figure 3. Related to Fig 2
(a) Primary T cells expressing Vβ subunits of interest were enriched using anti-TCRVβ-subunit antibodies and magnetic beads. (b) JRT3-T3.5 cells were transduced with lentiviral expression vectors encoding TCRβ chains and GFP. Cells were sorted to enrich cells which expressed CD3 on the cell surface. One week post sorting, cells were stained with antibodies specific for CD3 and the relevant TCRVβ subunit and analysed by flow cytometry. Fluorescence minus one controls were used to set the gates. (C) Histograms comparing the staining intensity of anti-TCRVB1, 2 and 9 PE-conjugated antibodies on primary T cell lines (red) and JRT3-T3.5 cells transduced with TCRβ chains (green).

## Slide 5
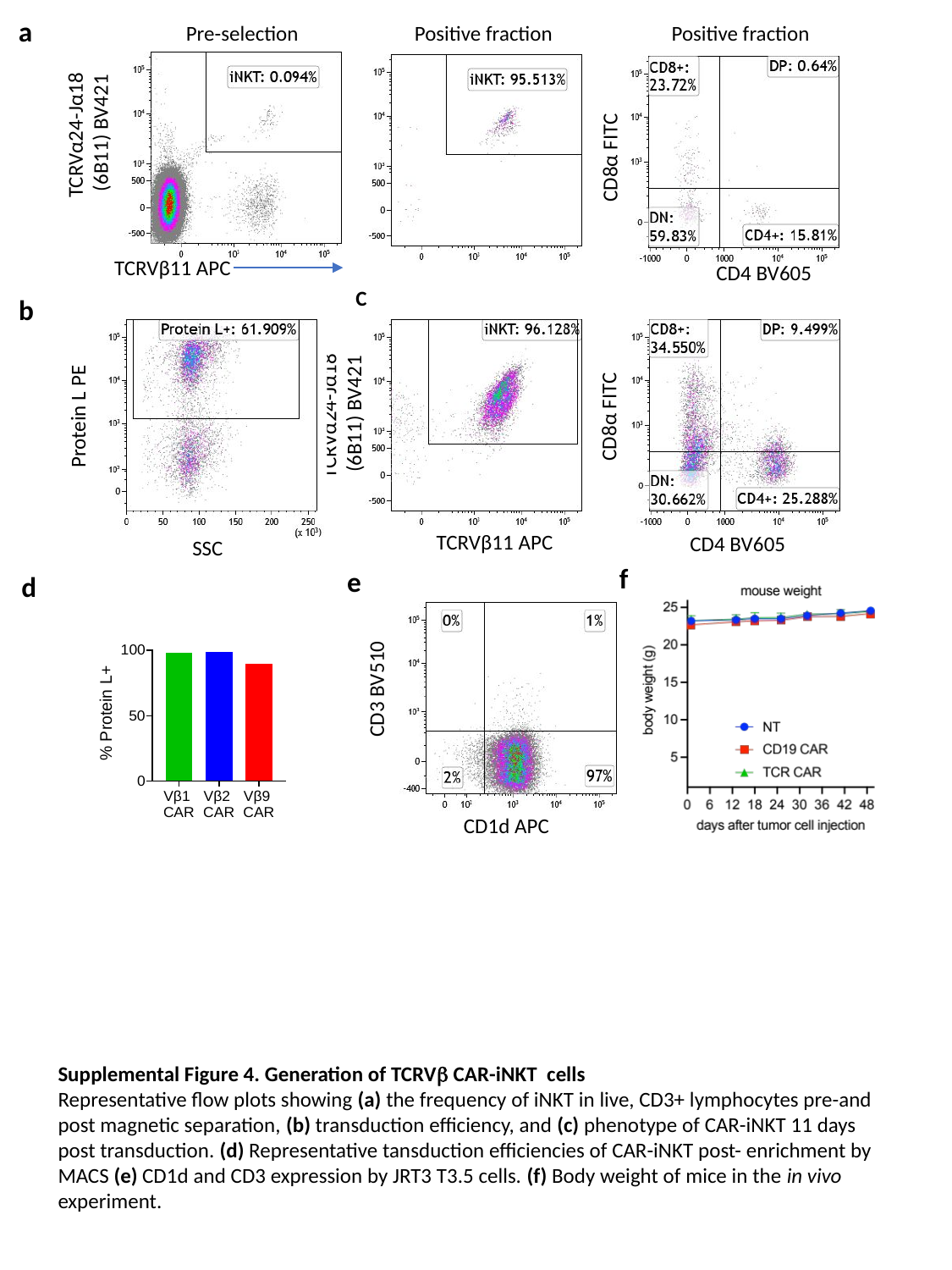

a
Positive fraction
Positive fraction
Pre-selection
TCRVα24-Jα18
(6B11) BV421
CD8α FITC
TCRVβ11 APC
CD4 BV605
C
b
TCRVα24-Jα18
(6B11) BV421
Protein L PE
CD8α FITC
TCRVβ11 APC
CD4 BV605
SSC
f
e
d
CD3 BV510
CD1d APC
Supplemental Figure 4. Generation of TCRVb CAR-iNKT cells
Representative flow plots showing (a) the frequency of iNKT in live, CD3+ lymphocytes pre-and post magnetic separation, (b) transduction efficiency, and (c) phenotype of CAR-iNKT 11 days post transduction. (d) Representative tansduction efficiencies of CAR-iNKT post- enrichment by MACS (e) CD1d and CD3 expression by JRT3 T3.5 cells. (f) Body weight of mice in the in vivo experiment.

## Slide 6
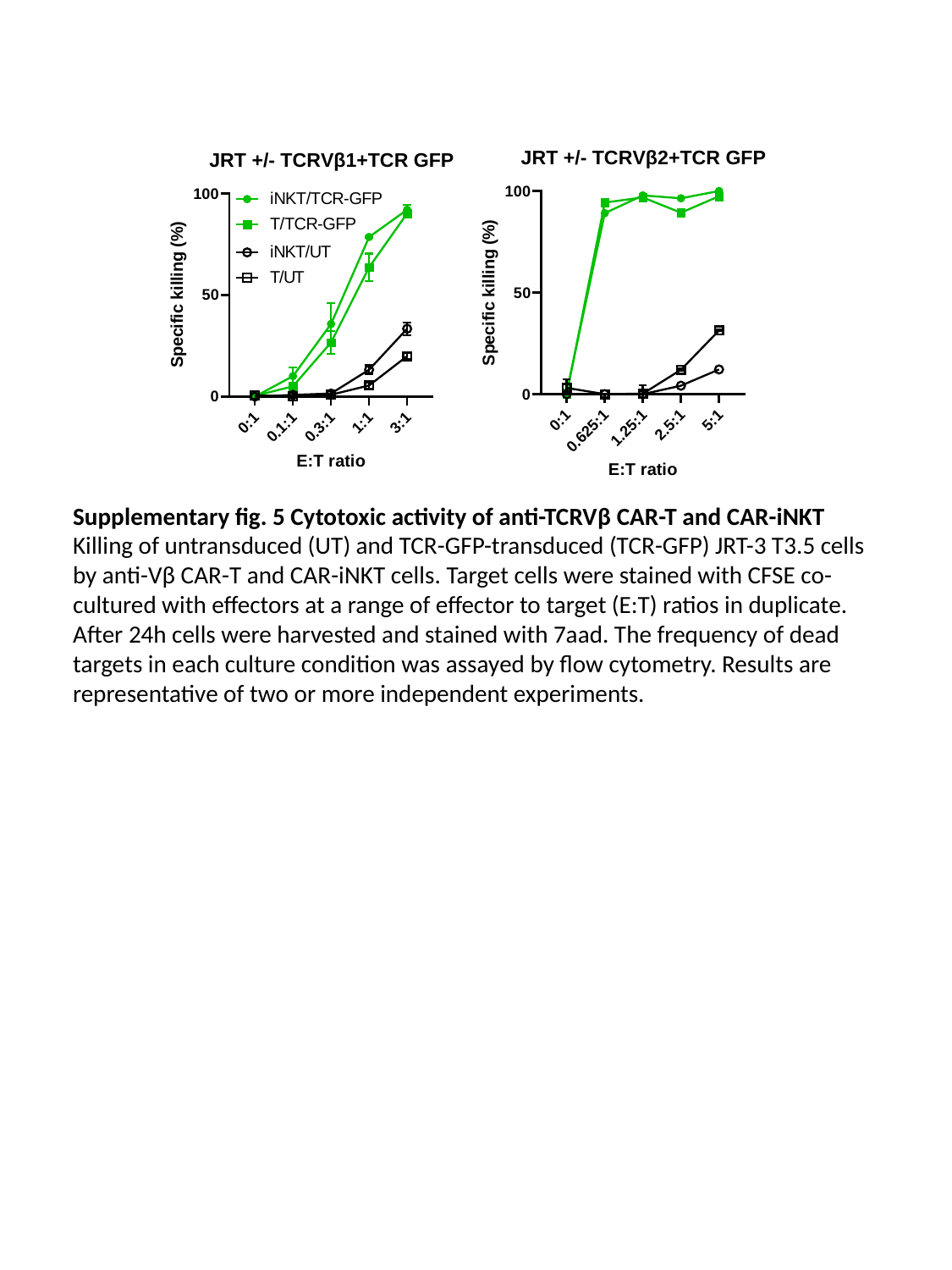

Supplementary fig. 5 Cytotoxic activity of anti-TCRVβ CAR-T and CAR-iNKT
Killing of untransduced (UT) and TCR-GFP-transduced (TCR-GFP) JRT-3 T3.5 cells by anti-Vβ CAR-T and CAR-iNKT cells. Target cells were stained with CFSE co-cultured with effectors at a range of effector to target (E:T) ratios in duplicate. After 24h cells were harvested and stained with 7aad. The frequency of dead targets in each culture condition was assayed by flow cytometry. Results are representative of two or more independent experiments.

## Slide 7
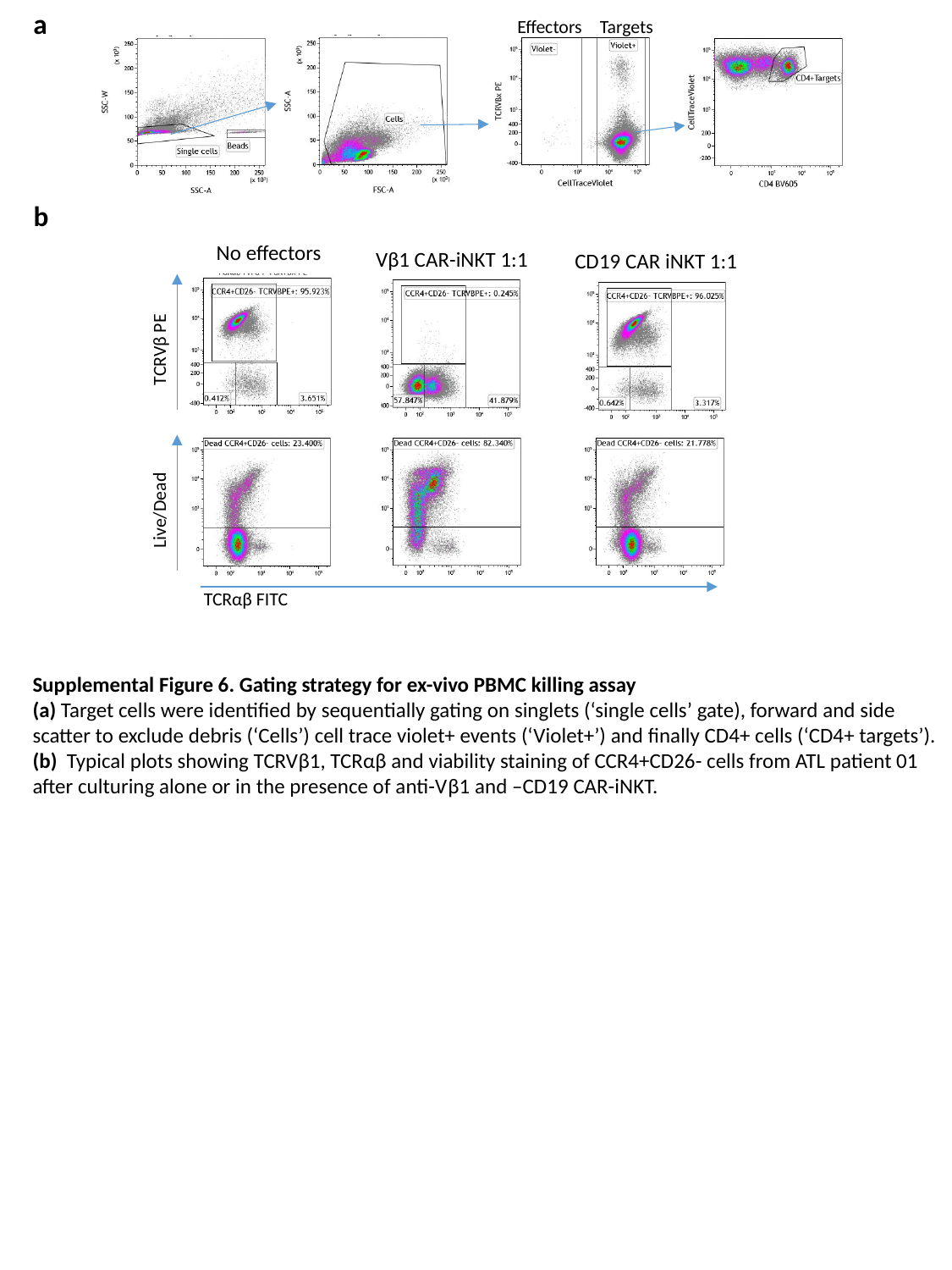

a
Targets
Effectors
b
No effectors
Vβ1 CAR-iNKT 1:1
CD19 CAR iNKT 1:1
TCRVβ PE
Live/Dead
TCRαβ FITC
Supplemental Figure 6. Gating strategy for ex-vivo PBMC killing assay
(a) Target cells were identified by sequentially gating on singlets (‘single cells’ gate), forward and side scatter to exclude debris (‘Cells’) cell trace violet+ events (‘Violet+’) and finally CD4+ cells (‘CD4+ targets’). (b) Typical plots showing TCRVβ1, TCRαβ and viability staining of CCR4+CD26- cells from ATL patient 01 after culturing alone or in the presence of anti-Vβ1 and –CD19 CAR-iNKT.

## Slide 8
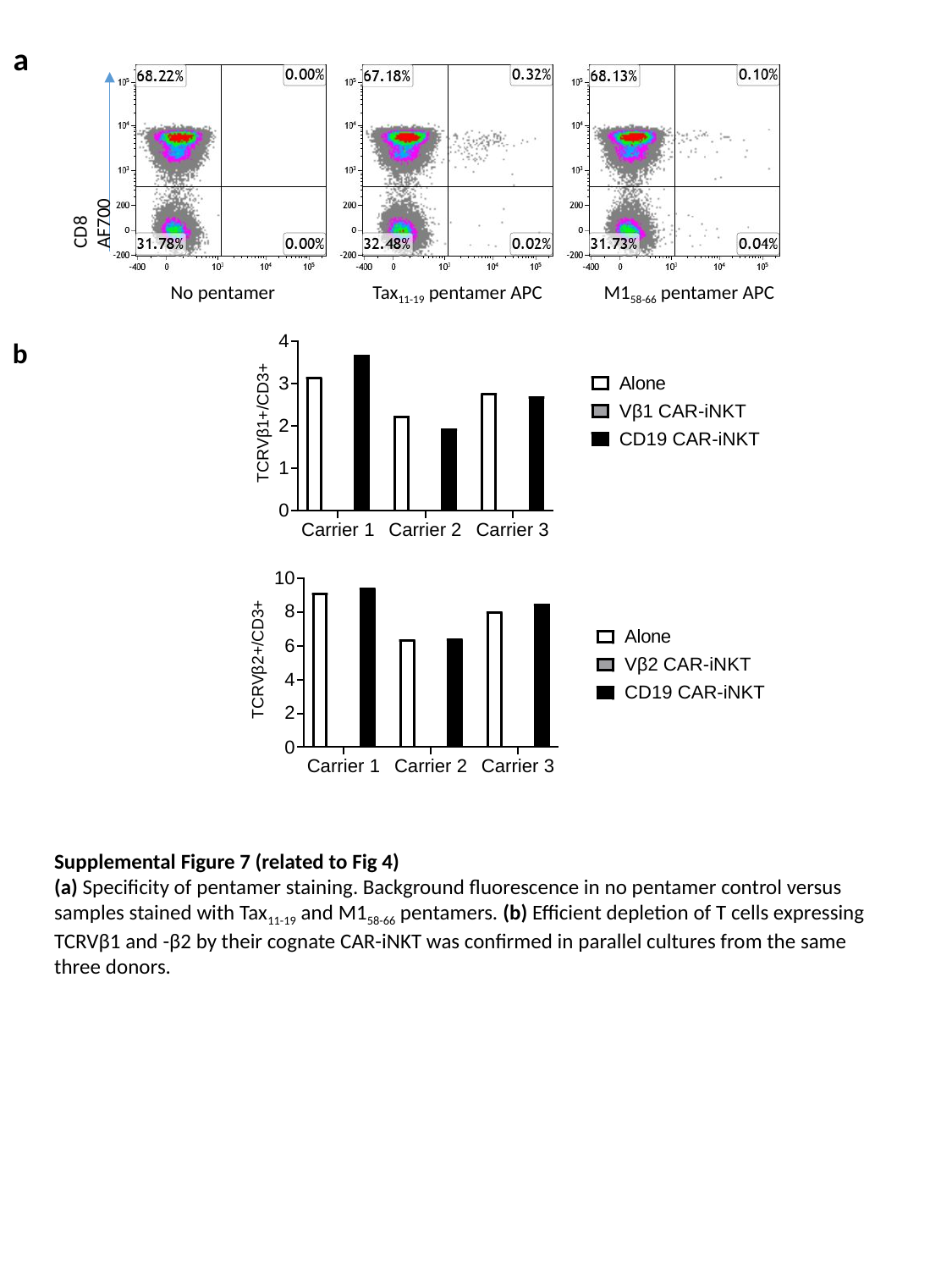

a
CD8 AF700
No pentamer
Tax11-19 pentamer APC
M158-66 pentamer APC
b
Supplemental Figure 7 (related to Fig 4)
(a) Specificity of pentamer staining. Background fluorescence in no pentamer control versus samples stained with Tax11-19 and M158-66 pentamers. (b) Efficient depletion of T cells expressing TCRVβ1 and -β2 by their cognate CAR-iNKT was confirmed in parallel cultures from the same three donors.
